# Emergency department consultations for respiratory symptoms revisited: exploratory investigation of longitudinal trends in patients’ perspective on care, health care utilization, and general and mental health, from a multicenter study in Berlin, Germany

**DOI:** 10.1186/s12913-022-07591-5

**Published:** 2022-02-10

**Authors:** Felix Holzinger, Sarah Oslislo, Lisa Kümpel, Rebecca Resendiz Cantu, Martin Möckel, Christoph Heintze

**Affiliations:** 1 Charité – Universitätsmedizin Berlin, corporate member of Freie Universität Berlin and Humboldt-Universität zu Berlin, Institute of General Practice, Charitéplatz 1, 10117 Berlin, Germany; 2 Charité – Universitätsmedizin Berlin, corporate member of Freie Universität Berlin and Humboldt-Universität zu Berlin, Division of Emergency Medicine Campus Mitte and Virchow, Charitéplatz 1, Berlin, 10117 Germany

**Keywords:** Emergency department, Follow-up, Patient satisfaction, Health care utilization, Respiratory conditions

## Abstract

**Background:**

Only few studies of emergency department (ED) consulters include a longitudinal investigation. The EMACROSS study had surveyed 472 respiratory patients in eight inner-city EDs in Berlin in 2017/2018 for demographic, medical and consultation-related characteristics. This paper presents the results of a follow-up survey at a median of 95 days post-discharge. We aimed to explore the post hoc assessment of ED care and identify potential longitudinal trends.

**Methods:**

The follow-up survey included items on satisfaction with care received, benefit from the ED visit, potential alternative care, health care utilization, mental and general health, and general life satisfaction. Univariable between-subject and within-subject statistical comparisons were conducted. Logistic regression was performed for multivariable investigations of determinants of dropout and of retrospectively rating the ED visit as beneficial.

**Results:**

Follow-up data was available for 329 patients. Participants of lower education status, migrants, and tourists were more likely to drop out. Having a general practitioner (GP), multimorbidity, and higher general life satisfaction were determinants of response. Retrospective satisfaction ratings were high with no marked longitudinal changes and waiting times as the most frequent reason for dissatisfaction. Retrospective assessment of the visit as beneficial was positively associated with male sex, diagnoses of pneumonia and respiratory failure, and self-referral. Concerning primary care as a viable alternative, judgment at the time of the ED visit and at follow-up did not differ significantly. Health care utilization post-discharge increased for GPs and pulmonologists. Self-reported general health and PHQ-4 anxiety scores were significantly improved at follow-up, while general life satisfaction for the overall sample was unchanged.

**Conclusions:**

Most patients retrospectively assess the ED visit as satisfactory and beneficial. Possible sex differences in perception of care and its outcomes should be further investigated. Conceivable efforts at diversion of ED utilizers to primary care should consider patients’ views regarding acceptable alternatives, which appear relatively independent of situational factors. Representativeness of results is restricted by the study focus on respiratory symptoms, the limited sample size, and the attrition rate.

**Trial registration:**

German Clinical Trials Register (DRKS00011930); date: 2017/04/25.

**Supplementary Information:**

The online version contains supplementary material available at 10.1186/s12913-022-07591-5.

## Background

Emergency departments (ED) are designated to provide care for urgent and serious conditions. Rising numbers of ED visits are reported from many international health care settings [1, 2], and consultations by patients with supposedly less urgent symptoms are frequently discussed as a booster of utilization in this context [3, 4]. Underlying reasons are multi-faceted, including issues of health literacy, health-related anxiety, and access barriers in primary care [5–7]. In this complex debate, directing blame on presumedly irresponsible patients is oversimplifying and inexpedient [8].

The mixed-methods EMACROSS (Emergency and Acute Care for Respiratory Diseases beyond Sectoral Separation) study investigated ED utilization in patients with respiratory complaints. Such are a frequent consultation trigger, and causes encompass a broad spectrum of serious (e.g. respiratory failure, pneumonia) as well as more trivial (e.g. upper respiratory tract infections) conditions. The main study module was a multicenter survey of 472 ED consulters [9], and baseline data (at index ED visit) showed a mixed picture: on the one hand, half of the patients in the cohort had a chronic pulmonary condition, a third reported repeated ED visits, and respiratory failure was diagnosed in ~ 20%. On the other hand, about 40% were triaged in lower urgency MTS (Manchester Triage System) categories 4 or 5, and more than 60% were treated as ED outpatients. The subgroup of self-referred walk-in patients showed lesser acute and chronic morbidity, and we could identify determinants of such consultations, including younger age and having no regular attachment to a general practitioner (GP). Concerning consultation motives, personal distress and access problems in ambulatory care were stated most frequently, while convenience reasons appeared of comparably minor importance. The results thus seemed not to indicate prevalent careless utilization of EDs as an easy primary care substitute. The situation is similar for patients arriving with emergency medical services (EMS): in a subgroup investigation published recently [10], the proportion of cases qualifying as potential primary care patients was quite low, particularly if considering patients’ views of the adequate setting. In the EMACROSS baseline survey, only 23% of patients had considered a GP visit a viable alternative to ED care.

However, our data was collected at the time of ED presentation, patients’ judgment being presumably influenced by the complaints’ frequent association with situational distress and anxiety. This potentially applies to all facets of patients’ appraisal of their ED visit: necessity and benefit of treatment received, satisfaction with care, etc. Whether patients’ views on ED consultations remain constant in the longer term or do change with timely distance to the event thus is an interesting question. Reports on consulters’ post hoc perspective on ED visits from survey studies are scarce. Longitudinal quantitative studies mainly focus on disease-specific course and prognostic impact, or health care utilization measures (e.g. [11–14]). Qualitative publications have investigated patients’ views on their consultations in-depth [6, 8, 15] and are very helpful to understand motives and decision-making. Nevertheless, they have no longitudinal approach and samples are inherently small.

The three-month follow-up survey presented in this paper therefore investigated how respiratory patients retrospectively assess their ED consultation. The investigations were conducted with an explorative intent and altogether had pilot character. We aimed to explore whether patients are content with the visit when reviewing it with temporal distance. Additionally, we looked at personal health care utilization and self-reported general health and life satisfaction after the event as compared to the baseline, as potential indicators of the ED visits’ consequences. A further research aim was the investigation of dropout determinants by comparison of follow-up responders to non-responders.

## Methods

Study design and conduct of EMACROSS have been described in detail in a previous publication on the baseline data [9]. To give readers a condensed overview, we outline the principal study features in the following sections and provide details on collection of the follow-up data presented in this article.

### Research network, setting, and recruitment

EMACROSS was conducted within the Berlin-based network EMANet (Emergency and Acute Medicine Network for Health Care Research Berlin) [16], which investigates acute care in eight hospital EDs in Berlin’s central district, including two university medical centers. Its focus was on ED consulters with respiratory symptoms. Recruitment was carried out between 1st of June 2017 and 30th of November 2018. Patients were eligible if presenting with a respiratory complaint (e.g. cough, dyspnea, wheeze etc.), fit to give written informed consent, and proficient in one of the survey languages. Recruitment focus was placed on regular physicians’ office hours as to our interest in choosing the ED versus conceivable alternative care, supplemented by intermittent eligibility screening off-hours (weekend, evenings) guided by a general target corridor rather than a strict pre-defined schedule.

### Data collection

All participants took part in the main study module, a quantitative survey with a tablet-based 43-item questionnaire (see supplement to [9]). Qualitative semi-structured interviews were additionally conducted with a subsample [17]. Secondary data from hospital records provided supplementary information, e.g. concerning ED and hospital diagnoses. Baseline survey participants were contacted again three months after the index ED visit by study personnel for a follow-up interview. This was preferably conducted by phone, but patients could opt for a written questionnaire if they preferred this. If patients could repeatedly not be reached by phone, they were likewise mailed a paper-based questionnaire. To maximize follow-up yield, municipal resident register information was obtained if participants could repeatedly not be reached, to ascertain whether the participants changed address or died since the index ED visit. Active follow-up efforts were concluded if unsuccessful after six weeks; written questionnaires returned at any later date were not discarded to minimize information loss.

The follow-up survey included items on satisfaction with care received in the ED (5-point Likert scale) and on whether patients had benefited from the ED visit in their own view (dichotomous item, yes/no). If applicable, reasons for dissatisfaction with care as well as for assessing the visit as beneficial were inquired. A dichotomous question on whether general practitioner treatment would have been a viable alternative was also included to capture self-assessment of the ED visits’ necessity. The baseline survey had also comprised this item. Other questions covered patients’ health care utilization (HCU) after discharge from the ED (or from an ensuing inpatient stay). Further items on general and mental health (see section “Variable definitions, data preparation” below) were adopted from the baseline assessment [9] to investigate eventual longitudinal changes. Two versions of the questionnaire were used for inpatients and outpatients, with questions and phrasing slightly adapted to fit the care level received. Some questions, e.g. concerning reasons for not being satisfied or for appraising the ED visit as beneficial, were posed openly and matched by study personnel to a list of pre-formulated options, to allow participants to more freely relate their views. Mentioned aspects not represented by this catalogue were documented in free text. In case of written questionnaires, respective items were posed as multiple-choice with the additional option of adding reasons not listed.

### Variable definitions, data preparation

Demographic and medical characteristics of the cohort were assessed at study baseline (index ED visit), either from the survey or from ED and hospital records. For variable definitions and data preparation of this dataset, we refer to the detailed outline in our previous publication [9]. In the three-month follow-up survey, a number of variables were defined and assessed identically to the baseline survey: general health with a 100-point visual analogue scale (0 = worst imaginable health, 100 = best imaginable health), mental health with the PHQ-4 instrument (possible scores of 0–12 and sub-scores of 0–6 for anxiety and depression) [18], general life satisfaction with the short scale L1 (0–10 point Likert scale; 0 = not satisfied at all, 10 = very satisfied) [19]. HCU questions were adapted from the German Health Interview and Examination Survey for Adults (DEGS) [20] and assessed consultation frequencies of GPs, medical specialists, emergency departments, inpatient care, and urgent primary care on-call services, for the period since the index visit. The abovementioned scales and tools represent validated and established instruments (PHQ-4, short scale L1, general health visual analogue scale, HCU assessment), while the remainder of the survey items was newly developed on a theoretical basis. To compare HCU data collected at follow-up to the baseline survey, in which consultation frequencies had been assessed for a retrospective six months, corresponding utilization was calculated for a three-month period (90 days). For baseline data, this meant halving six-month numbers, while for follow-up data, the individual time between index visit and follow-up was considered. This approach is equivalent in principle to commonly performed standardizations of HCU data to uniform time frames (e.g. annualization) [21]. For some questions exclusive to the follow-up survey (e.g. reasons for non-satisfaction, or for rating the visit as beneficial), multiple answers were allowed, resulting in multi-response data. As to missing data, it was decided to refrain from using statistical techniques like multiple imputation, as missingness was not of a major dimension. Neither could we be sure whether the missing at random assumption applied.

### Data analysis

Data from questions exclusive to the follow-up survey was analyzed and summarized descriptively. Cross-sectional group comparisons (= between-subject comparisons) were conducted with the χ^2^ test for categorical and the Mann-Whitney-U-test for continuous variables. For correlation of binary variables, Pearson’s phi coefficient was used.

Determinants of dropout and of patients rating the ED visit as beneficial were investigated by logistic regression. The aim was to identify contributive – and potentially explanatory – factors and explore their impact on the outcomes’ probability. The decision to investigate these two outcomes by multivariable analyses was taken for specific reasons. Concerning dropout determinants, a detailed investigation was deemed important as to its impact on the validity of the study results. As to patient assessment of the ED visit as beneficial (or not), this was considered an especially interesting – although explorative – outcome. For the regression analyses, a set of variables of interest as potential contributive factors or control variables was compiled and univariable statistics conducted (χ2 test for categorical and Mann-Whitney-U-test for continuous variables). Non-significance of variables did not result in compulsory rejection, variables being retained if e.g. deemed important from a theoretical viewpoint or as a control variable [22]. We constructed a preliminary multivariable model and observed the effects of discarding and re-adding variables. Models including different sets of variables were compared as to fit and accuracy by the Hosmer Lemeshow test, Nagelkerke’s R^2^, and AUC (area under the ROC curve) [23]. We did not use an automated variable selection method as to avoid associated model bias [24].

Longitudinal comparisons (= within-subject comparisons) were conducted with the McNemar test for categorical and the Wilcoxon signed-rank test for continuous variables. The significance level for all analyses was set at 0.05. Analyses were performed in SPSS Version 27 (IBM Corp., Armonk, NY, USA) and R 3.6.2 (R Foundation for Statistical Computing, Vienna, Austria).

## Results

### Follow-up yield and characteristics of responders vs. non-responders

Of the 472 patients in the baseline cohort, 329 could be reached by follow-up efforts. Interviews were conducted with 266 participants, while 63 returned a printed questionnaire. The dropout rate was 30.3%, with Table 1 showing reasons for participants being lost to follow-up. We know of 15 patients (3.18%) who died in the period between index visit and intended follow-up.

**Table 1:** Yield of follow-up efforts and reasons for dropout

Patients	n	For lost to follow-up: % of 143 patients
**Baseline survey**	472	–
**Follow-up survey**	329	–
**Lost to follow-up**	143	100.0
Died	15	10.5
Could not be reached: moved with no forwarding address obtainable (incl. to other countries), invalid phone/e-mail, or not answering at multiple contact attempts	73	51.0
Patient reached and consented to follow-up, but did not return questionnaire	19	13.3
Patient reached, but refused to participate	36	25.2
Most frequent reasons stated:		
Language barrier	12	8.4
Time constraints	6	4.2
Health status	6	4.2

Median follow-up time (distance to baseline) was 95 days, with a range of 82 to 206 days. Most patients were followed up at three to four months after the index visit, with the 95th percentile of follow-up length at 133 days.

Table 2 summarizes basic demographic and medical characteristics of the total baseline cohort and the subgroup of patients with available follow-up.

**Table 2:** Data from baseline survey: characteristics of total cohort, patients with follow-up data available, follow-up non-participants (total), patients who died prior to intended follow-up, and follow-up non-participants excluding patients who died

Variable	Measure	Total cohort	Follow-up sub-cohort	Follow-up non-participants (total)	Follow-up non-participants who died	Follow-up non-participants, excl. patients who died
**Participants**	n	472	329	143	15	128
***Demographics***						
**Age**	n	472	329	143	15	128
	Mean (SD)	53.6 (19.2)	54.6 (18.3)	51.3 (21.0)	79.7 (7.1)	48.0 (19.5)
	Median (Range)	55.0 (18–96)	56.0 (18–92)	51.0 (18–96)	81.0 (67–92)	46.0 (18–96)
**Sex**	n	472	329	143	15	128
Male	%	53.2	52.9	53.8	73.3	51.6
Female	%	46.8	47.1	46.2	26.7	48.4
**Migration and travel**	n	466	326	140	15	125
Migrant first generation	%	21.9	17.2	32.9	6.7	36.0
Second generation	%	6.9	6.1	8.6	0.0	9.6
Tourist	%	4.3	1.2	11.4	0.0	12.8
**Education (CASMIN)**	n	463	325	138	15	123
Low	%	25.5	23.7	29.7	73.3	24.4
Intermediate	%	43.6	46.8	36.2	20.0	38.2
High	%	30.9	29.5	34.1	6.7	37.4
***ED consultation***						
**Means of arrival**	n	462	319	143	15	128
Walk-in	%	63.0	60.2	69.2	26.7	74.2
EMS	%	30.7	31.7	28.7	73.3	23.4
Ambulance transport	%	6.3	8.2	2.1	0.0	2.3
**Initiation of visit**	n	465	325	140	15	125
Self-referred	%	62.8	59.4	70.7	60.0	72.0
Health professional	%	37.2	40.6	29.3	40.0	28.0
**Triage category**	n	456	317	139	14	125
Lower urgency	%	41.9	40.4	45.3	0.0	50.4
Higher urgency	%	58.1	59.6	54.7	100.0	49.6
**Time of presentation**	n	472	329	143	15	128
Out-of-hours visit	%	17.2	16.7	18.2	33.3	16.4
During office hours	%	82.8	83.3	81.8	66.7	83.6
***ED symptoms***						
**Symptom novelty**	n	467	326	141	15	126
New symptoms	%	36.4	37.7	33.3	0.0	37.3
Recurrent symptoms	%	63.6	62.3	66.7	100.0	62.7
**Symptom-associated distress**	n	442	312	130		
	Mean (SD)	7.2 (1.8)	7.3 (1.8)	7.0 (1.9)	7.4 (1.9)	6.9 (1.9)
	Median (Range)	7.5 (1.5–10)	7.5 (2.0–10)	7.0 (1.5–10)	8.0 (3.5–9.5)	7.0 (1.5–10)
***Chronic conditions and care***						
**Chronic pulmonary condition**	n	467	326	141	15	126
	yes: %	58.7	62.0	51.1	66.7	49.2
**Multimorbidity**	n	465	325	140	15	125
	yes: %	53.5	58.8	41.4	73.3	37.6
**Attached to GP**	n	464	323	141	15	126
	yes: %	86.6	90.7	77.3	100.0	74.6
***Mental and general health***						
**PHQ-4 anxiety subscale**	n	467	326	141	15	126
	Mean (SD)	1.7 (1.9)	1.6 (1.8)	2.0 (2.0)	1.8 (2.1)	2.0 (2.0)
	Median (Range)	1.0 (0–6)	1.0 (0–6)	1.0 (0–6)	1.0 (0–6)	1.0 (0–6)
**PHQ-4 depression subscale**	n	467	326	141	15	126
	Mean (SD)	2.2 (2.2)	2.1 (2.1)	2.4 (2.3)	2.7 (2.3)	2.3 (2.3)
	Median (Range)	2.0 (0–6)	2.0 (0–6)	2.0 (0–6)	3.0 (0–6)	2.0 (0–6)
**General life satisfaction**	n	457	321	136	15	121
	Mean (SD)	6.9 (2.6)	7.1 (2.5)	6.7 (2.8)	6.4 (3.1)	6.7 (2.8)
	Median (Range)	8.0 (0–10)	8.0 (0–10)	8.0 (0–10)	8.0 (0–10)	8.0 (0–10)
**General health**	n	466	325	141	15	126
	Mean (SD)	45.9 (25.0)	45.8 (24.6)	46.1 (25.9)	33.2 (23.3)	47.6 (25.8)
	Median (Range)	50.0 (0–100)	50.0 (0–100)	50.0 (0–100)	40.0 (0–80.0)	50.0 (0–100)
***ED visit outcomes***						
**Diagnoses**	n	472	329	143	15	128
Pneumonia J12-J18	%	23.3	25.8	17.5	26.7	16.4
COPD and chronic bronchitis J40-J44	%	34.3	38.3	25.2	60.0	21.1
Asthma bronchiale J45-J46	%	9.7	8.8	11.9	0.0	13.3
Other respiratory tract infection J09-J11, J20-J22	%	8.5	8.5	8.4	0.0	9.4
Upper airway conditions J0x/J3x	%	10.2	8.2	14.7	0.0	16.4
Respiratory symptom diagnosis only (R section code)	%	14.4	14.0	15.4	13.3	15.6
Respiratory failure J96	%	19.5	20.7	16.8	46.7	13.3
**Visit consequence**	n	472	329	143	15	128
Outpatients	%	61.2	58.4	67.8	20.0	73.4
Hospital admission	%	38.8	41.6	32.2	80.0	26.6

*n* Cases with available data for respective characteristic, *%* Percentage of cases with available data; Ranges reported with median values refer to minimum and maximum; Migration and travel: first generation = not born in Germany, second generation = participant born in Germany and mother/father (or both) born in another country; Education: *CASMIN* Comparative Analysis of Social Mobility in Industrial Nations scale, trichotomized; Triage category: MTS categories 4 and 5 = lower urgency, categories 1,2 and 3 = higher urgency; Time of presentation: out-of-hours defined as between 6 pm and 8 am on weekdays, plus weekends and Wednesdays afternoons after 2 pm; Subjective symptom-associated distress, general life satisfaction: 0–10 scales; Chronic pulmonary condition: if either self-reported or documented in hospital records; Multimorbidity = two or more self-reported chronic conditions; PHQ-4 anxiety and depression: 0–6 subscales; General health: 0–100 visual analogue scale; Diagnoses: ICD-10 codes, multiple diagnoses possible for individual cases

In the regression model of dropout determinants (434 cases, Nagelkerke’s R^2^ 0.232, AUC 0.75, Hosmer-Lemshow test χ^2^ 10.341, df = 8, *p* = 0.242), lower probabilities of response to follow-up could be seen for first-generation migrants (OR 0.340, 95% CI [0193;0.599], *p* < 0.001, reference category: no migration and travel characteristic present) and tourists (OR 0.033, 95% CI [0.009;0.128], *p* < 0.001, same reference category). Patients with high educational status (OR 2.042, 95% CI [1.035;4.032], *p* = 0.040, reference category: low educational status) and multimorbidity (OR 1.947, 95% CI [1.136;3.338], *p* = 0.015) showed higher likelihood to take part in the follow-up survey. This was also the case for patients reporting to have a GP (OR 2.296, 95% CI [1.187;4.441], *p* = 0.014), and patients with higher general life satisfaction ratings (OR 1.125, 95% CI [1.029;1.231], *p* = 0.010). Sex and age were included in the model as control variables.

A closer look at the 15 patients who reportedly died prior to intended follow-up shows that these were of high age (mean ~ 80 years) and predominantly male. Morbidity indicators (e.g. urgent triage categories, high share of chronic illness, respiratory failure diagnosed in nearly half, high rate of hospital admission) show that many were already very ill at the time of the index ED visit. It must be noted that death during the designated follow-up period was confirmed in these cases either by relatives reached by our contact efforts, or official municipal register information. Causes or manner of death were not investigated. For some patients (who e.g. moved to another country), no such data was available, so we cannot be absolutely certain that all patients listed in Table 1 as “could not be reached” were still alive three months after the ED index visit.

### Post hoc assessment of the ED visit

Retrospective satisfaction ratings regarding the ED visit were high, with 79.7% (of 325 patients with data for this item) of affirmative answers on the 5-point Likert scale (ratings “very satisfied” or “satisfied”). Of the 66 patients who did not rate satisfaction in favorable categories, reasons for not being satisfied were inquired (multiple answers possible). Most frequent complaints were about waiting times (*n* = 42, 63.6%), symptoms not being taken seriously (*n* = 15, 22.7%), insufficient treatment for acute complaint (*n* = 16, 24.2%), unfriendly staff (*n* = 11, 16.7%) and lack of information regarding treatment (*n* = 8, 12.1%). Distribution of the dissatisfaction reasons stated did differ between the sexes. Symptoms not being taken seriously were criticized by 17.2% of men vs. 27.0% of women, insufficient treatment by 20.7% of men vs. 27.0% of women, unfriendly staff was reported by 20.7% of men vs. 13.5% of women, and lack of information received by 6.9% of men and 16.2% of women. As to waiting times there was no marked difference (62.1% in men, 64.9% in women).

In the index visit survey, patients had also been asked to rate their satisfaction with current ED treatment in a Likert-scaled item corresponding to the follow-up question. Of 281 follow-up patients with a respective baseline rating available, 87.9% had then selected “very satisfied” or “satisfied”, and rating in a thus combined category was concordant to the retrospective assessment at follow-up in 216 cases (76.9%). There were no significant longitudinal differences for satisfaction ratings if assessed by Wilcoxon signed-rank test (Likert scale interpreted as quasi-continuous, *p* = 0.432) or McNemar test (scale dichotomized in top two vs. other categories, *p* = 0.120). Data did not show a marked difference between consulters coming to the ED during office hours vs. off-hours (*p* = 0.423, Mann-Whitney-U-test, 325 cases).

Two hundred fifty-eight participants (81.4% of 317 patients with data for this item) stated that they had benefited from their ED visit. When inquired about reasons for assessing the ED visit as beneficial, aspects reported by 256 cases included the initiation of a necessary inpatient or outpatient treatment (50.4%), personal reassurance (46.1%), prevention of aggravation by timely intervention (47.3%), the detection of the complaints’ cause (40.6%) and feeling relieved (35.2%). Assessment of the ED visit as beneficial was moderately correlated with satisfaction (binarized to “very satisfied” / “satisfied” categories vs. other categories), with a phi coefficient of 0.52 (*p* < 0.001).

Univariable statistics indicated that patients with a pneumonia diagnosis did retrospectively assess the ED consultation significantly more frequently as beneficial (*p* = 0.010), as well as patients with respiratory failure (*p* = 0.001), while this was not the case for COPD, Asthma, RTI, or Upper Airway diagnoses. A rating of the visit as beneficial was also significantly more frequent in male patients (*p* = 0.001), patients triaged as of high urgency (*p* = 0.026) and self-referrers (*p* = 0.025). Male sex, pneumonia, respiratory failure, and self-referral were confirmed as determinants of retrospectively assessing the ED visit as beneficial in a subsequent multivariable logistic model controlling for demographics (age, education, and migration). Triage was no longer significant in the multivariable analysis (Table 3). An additional graphical representation of the regression results is included in the Additional file 1 (Supplementary Fig. 1).

**Table 3:** Logistic regression model for rating ED visit as beneficial

Rating of ED visit as beneficial, 304 complete cases							
Independent variable		Coefficient B	Standard error	*p* value	Odds ratio	OR 95% CI lower bound	OR 95% CI upper bound
**Age**		−0.011	0.010	0.249	0.989	0.970	1.008
**Sex** (male)		0.917	0.335	0.006	2.501	1.298	4.819
**Pneumonia**		1.079	0.458	0.018	2.941	1.199	7.214
**Respiratory failure**		1.542	0.638	0.016	4.676	1.338	16.342
**Self-referral**		0.854	0.344	0.013	2.350	1.197	4.613
**Triage** (higher urgency)		0.305	0.335	0.363	1.357	0.703	2.619
**Education**^**a**^	-intermediate	−0.160	0.450	0.721	0.852	0.353	2.056
	-high	−0.252	0.481	0.600	0.777	0.303	1.996
**Migrant**^**b**^		−0.303	0.418	0.469	0.739	0.326	1.676

Model performance metrics: AUC 0.74; Nagelkerke’s *R*^2^ 0.17; Hosmer-Lemeshow test χ^2^ = 7.302, df = 8, *p* = 0.504

^a^Education: CASMIN-scale, trichotomized, reference category: low

^b^Migrant: first generation (not born in Germany)

Further morbidity markers (diagnoses of asthma, RTI, upper respiratory conditions, presence of a chronic pulmonary condition), as well as variables on out-of-hours consultation, symptom-associated distress at baseline, and attachment to a GP, were also evaluated for the multivariable model, but did not show themselves relevant contributive factors.

We also explored possible associations of the manner of follow-up response (telephone vs. writing) regarding ratings of satisfaction and subjective benefit from the ED visit. As to satisfaction, there were differences: in the telephone group, 82.5% rated satisfaction in the two top categories, while only 67.7% did so in written questionnaires (*p* = 0.009, χ2 test). This did not apply to rating the ED visit as beneficial, where proportions corresponded (81.7 and 80.0%, *p* = 0.771). Data did neither suggest an influence of follow-up time on either outcome, which was explored by comparing patients followed up at up to 95 days (median follow-up time) and at more than 95 days.

When revisiting their ED consultation at follow-up, 66 participants (21.4% of 309 cases with available data) believed a GP could also have solved their problem. This corresponds to the overall assessment in the baseline survey, where 20.6% (of 277 cases followed up with baseline data available for this item) considered primary care a suitable alternative in their acute situation. There was no significant difference between time points (McNemar test *p* = 0.332, 260 cases).

### Health care utilization

For the time after the ED visit, 94.8% reported at least one contact with a health care provider or institution. Table 4 summarizes HCU during follow-up.

**Table 4:** Self-reported health care utilization during the follow-up period and pre-post-comparison (three months prior to / after ED index visit)

Provider / institution	n^a^	% Utilization during follow-up	Mean no of visits pre (SD)	Mean no of visits post (SD)	*p* for pre-post comparison^b^
**GP**	302	83.8	1.4 (1.3)	2.3 (2.6)	< 0.001
**Medical specialist**	288	51.4	1.3 (2.4)	1.5 (2.5)	0.829
**Pulmonologist**	301	47.2	0.4 (0.8)	0.8 (1.1)	< 0.001
**Emergency department**	296	28.0	0.3 (0.5)	0.4 (1.0)	0.118
**Emergency house call service**	293	7.2	0.1 (0.4)	0.2 (1.1)	0.676
	n^a^	% Utilization during follow-up	Mean total duration pre (SD), all patients	Mean total duration post (SD), all patients	*p* for pre-post comparison^b^
**Hospital inpatient stay**	302	34.4	2.5 (6.6)	3.7 (7.6)	0.006
	n^a^	% Utilization during follow-up	Mean total duration pre (SD), patients with inpatient stay^c^	Mean total duration post (SD), patients with inpatient stay^c^	*p* for pre-post comparison^b^
**Hospital inpatient stay**	104	100.0	5.2 (9.5)	10.9 (9.5)	< 0.001

HCU standardized to a three month period pre/post index visit. Mean duration of hospital stay in days, mean total duration refers to all stays reported by patient, excluding an eventual inpatient stay directly ensuing the index ED visit

^a^*n* = number of cases with baseline and follow-up HCU data available

^b^Wilcoxon signed-rank test

^c^Patients who had an inpatient stay between baseline and follow-up, excluding stays directly ensuing index visit

**Fig. 1:**
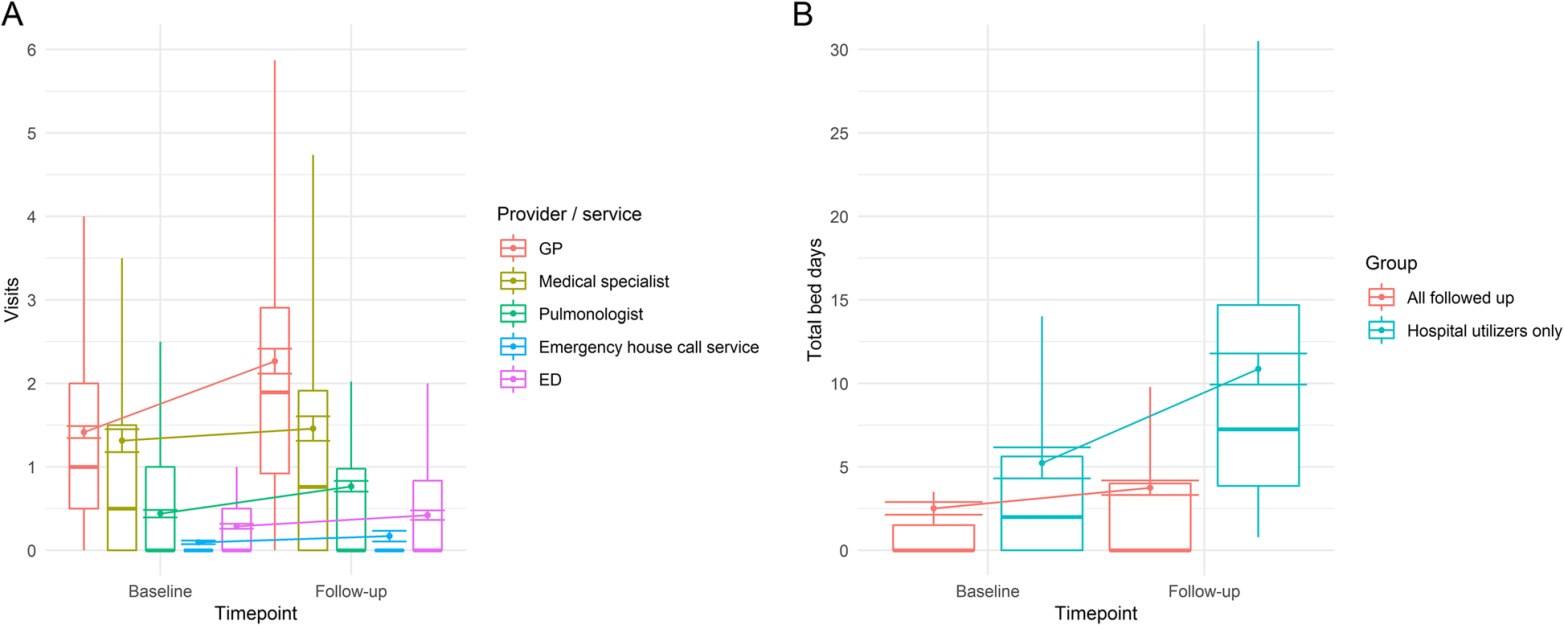
Retrospective three-month health care utilization assessed at both survey time points. **A** Provider / service visits, **B** Inpatient stays. *Note.* Boxes represent medians and quartiles, whiskers the largest observed point falling within distance of 1.5 interquartile range. Outliers are hidden to allow visual representation with reasonable scaling. Points represent means, error bars correspond to standard errors of means, and line chart elements show longitudinal trends

Compared to self-reported HCU in the baseline survey, increases were seen for GPs and pulmonologists, while longitudinal differences in ED and medical specialist consultations were not significant. The total of hospital inpatient days was also significantly higher in the period after the index ED visit, with mean numbers of hospital days almost doubled from pre-baseline for patients who had a hospital stay during the follow-up period. In the subgroup of hospital utilizers, ED utilization also showed a marked longitudinal increase (pre mean 0.5, SD 0.7; post mean 1.0, SD 1.5; *p* = 0.003). Figure 1 visualizes the longitudinal trends in HCU.

### General and mental health

Self-reported general health on the 100-point visual analogue scale improved significantly between baseline and follow-up (pre mean 45.7, SD 24.9; post mean 61.5, SD 24.9; Wilcoxon signed-rank test *p* < 0.001, 316 cases). General life satisfaction was not changed (*p* = 0.092, 308 cases). As to mental health, PHQ-4 anxiety and depression scores decreased over time (both *p* < 0.001, 309 and 313 cases). Detailed results for general and mental health outcomes can be found in the Additional file 1 (supplementary Table 1).

## Discussion

### Summary of findings

This study is one of very few quantitative longitudinal investigations focused on ED patients’ perspective rather than morbidity outcomes or re-admission determinants. Follow-up data was available for 329 patients at a median time of 95 days after baseline. Retrospective satisfaction ratings were high, waiting times constituting the most frequent reason for not being satisfied. Ratings did not significantly change from baseline. Male sex was indicated as a determinant of assessing the ED visit as beneficial by logistic regression, with pneumonia, respiratory failure, and self-referral as additional contributive factors. A corresponding share of participants at baseline and follow-up opined that a GP could have provided adequate alternative treatment, with no significant longitudinal difference.

HCU in the total follow-up cohort did increase for GPs and pulmonologists, while there was no longitudinal difference for ED consultations. Self-reported general health improved significantly between baseline and follow-up, as well as PHQ-4 scores. General life satisfaction was not changed.

Participants of lower educational status, migrants, and tourists were more likely to drop out of the study, while having a GP, as well as both multimorbidity and higher general life satisfaction, were independent determinants of response to the follow-up survey.

### Results in context

#### Post hoc assessment of the ED visit

The retrospective view on ED care and its consequences was assessed by more than one question in this survey. We opted for a combination of items to capture dimensions of what could be described as patients’ “happiness” or “contentedness” with an ED visit. Patients surveys differ from consumer satisfaction surveys, as they have to consider both the process and the outcomes of care, as has been discussed by e.g. Hudak et al. [25]. A concept of being “happy” with what happened at the ED and resulted from it thus includes organizational aspects (e.g. experiencing care as timely, friendly and professional), which was mainly captured by our items on satisfaction. However, it also encompasses the question of whether the consequences were positive health-wise: the visit should have contributed to improving health, or alleviating health-related concerns. This was aimed for by the question on “benefit” associated with the visit. Overall, we can derive from the items that only a minority of patients (^~^ 20%) is retrospectively discontent with an aspect of the ED visit, which is comparable in dimension to other investigations [26]. Regarding satisfaction, areas of criticism also correspond to core problems identified by previous studies, like waiting times or interpersonal behavior [27, 28].

Concerning determinants of a “beneficial” judgment, differences in self-referrers and patients with more severe conditions seem not so surprising, as these may subjectively profit the most, concerning either reassurance or intervention in an urgent health crisis. For critically ill patients, the outcome is key, and aspects of comfort and service lose importance, which has been described as “the point of view paradox” [29]. The sex differences seen are more puzzling to interpret: why do males rate the visits’ consequences more positively in retrospect? Literature on sex differences in assessment of emergency care mainly focuses on its role as a determinant of satisfaction, and results are conflicting: while some studies report higher service satisfaction in females [30, 31], many others did not show a difference [32, 33]. However, while sex is reported in most satisfaction evaluations and there is an abundancy of research on sex differences regarding ED treatment and outcome of specific conditions (including respiratory [34]), we could not identify any pertinent literature on sex-specific self-assessment of ED care as beneficial, or a related concept. Theoretically, the tendency of males in our sample to judge the benefit of care greater could be rooted in gender differences concerning perception of symptoms [35] or stress and anxiety [36], but this is certainly quite speculative. More concerningly, the results could indicate a genuine care gap with a tendency towards suboptimal care for women with respiratory complaints, as has been reported for cardiovascular conditions [37, 38]. Research suggesting a possible gender bias in e.g. diagnosing and managing COPD points in a similar direction, but these investigations were performed in a non-hospital setting [39, 40]. Differences in dissatisfaction reasons reported by men and women in our survey may also hint at women feeling undertreated and underinformed to a greater degree, with the obvious caveats of small patient numbers and a descriptive level. This issue clearly needs to be pursued in further studies.

The question of conceivable alternative care constitutes a further interesting facet of patients’ post hoc view on ED care. Interestingly, our study shows that this assessment does not change over time. This consistency suggests that patients’ judgment regarding the suitable care sector for a given situation is not markedly contorted by the acute experience of symptoms at the time of their ED consultation Conceivably, patients could have felt more urgent at the index visit than they might do in retrospect when revisiting the event, but this does not appear to be the case. While others have quantified proportions of non-urgent ED cases (e.g. [41]), with average reported rates amounting to more than 30% [5], it should be noted that we did not analyze patient data to delineate “real emergencies” vs. non-urgent cases, as it was not out aim to verify or disprove patient judgment. However, reasons why patients would prefer the ED go beyond medical considerations [8], and views might differ markedly from health professionals’ assessments [42, 43]. Results from the qualitative EMACROSS module have correspondingly illustrated the importance of patients’ self-assessment as genuine emergencies in urgent need of medical attention [44]. Looking at triage categories in our study, 57.1% of the patients stating that a GP would have been a viable alternative were initially triaged in non-urgent levels 4 and 5, as compared to only 35.0% in the group that retrospectively viewed the ED as without alternative (*p* = 0.001). This accredits patients’ judgments with a degree of validity.

#### Health care utilization

Considering that many ED patients receive the discharge recommendation to visit an ambulatory provider for follow-up checks or prescription of medications, the longitudinal rise in respective visits is not surprising. However, increasing effects on HCU triggered by medication changes associated with the ED visit (such were reported by a quarter) might be short-term and revert to earlier levels in the further course [45], which we do not know as to the relatively short follow-up period. Concerning this question, it would be instructive to know about the exact time points of post-discharge provider visits, but this would require respective administrative secondary data.

Neither is it clear whether HCU reports do indicate a prevailing decline in health after the ED visit. Supposedly, ED visits and emergency house calls would potentially have spiked as well if this were the case. The rise in hospital days, but not ED visits, seems to suggest that this may be mainly due to health problems requiring elective hospital treatment. However, a corresponding recommendation was reported by only ~ 8%, while the share of patients requiring hospital care between index visit and follow-up is considerably larger. Data also showed that, while not significantly changed in the overall cohort, ED utilization increased for the subgroup of patients with a hospital stay during follow-up. This hints at frequent non-elective hospital admissions via ED visits in this population.

This is interesting in connection with the discussion of ED visits’ role as a “sentinel event” for worsening health, which has been claimed especially for older and sicker populations and return visits [46, 47]. In our cohort comprising both younger and generally healthy persons as well as the aged and multimorbid, this is less pronounced than in studies with e.g. heart failure patients, for which repeat hospitalizations and ED visits are part of a typical trajectory of end-stage disease [48]. For our population, HCU data suggests that the notion of ED utilization as an early indicator of worsening health status is probably true for a share of patients, but not for the majority. It must be borne in mind however, that our data describes only patients surviving until they could be followed up, the 15 patients with the direst consequences not being represented. On the other hand, non-response to follow-up was greater for healthier participants, for which the ED visit might have been of minor impact.

#### General and mental health

A detailed analysis of general health and life satisfaction has been recently published for the entirety of the EMANet studies [49], of which EMACROSS represents one of three sub-projects. Self-reported health in ED patients across different symptom spectrums was found considerably lower compared to representative samples of German adults (visual analogue scale mean of ~ 77) [50], which corresponds to findings of poor self-reported health in other ED populations [51, 52]. Based on these results, it was concluded that ED visits indicate a serious health event with acute impact on well-being [49]. Our longitudinal data however shows tendential regression towards “average” population levels: acute events seemingly loose its repercussion over time. This is in line with findings from a longitudinal ED study of patients with lower respiratory tract infections and cardiac diseases, in which negative affects decreased during follow-up [53]. The striking longitudinal difference in self-reported general health suggests that baseline ratings probably rather reflect the acute event-related plight of the emergency than health status in general. The same could be the case for PHQ-4 scores, which are intended to cover anxiety and depression symptoms over the past two weeks, as opposed to symptom-related distress associated with the acute situation. Longitudinal trends give reason to suspect that the ED situation may have influenced judgment here. Thus, the PHQ-4, having been originally validated in a primary care setting [18], may be less suitable for assessing mental health in ED research, which should be considered when planning future studies. In our EMANet sister study EMASPOT focusing on patients with cardiac complaints and comorbid mental health conditions, the more extended PHQ-9 and GAD-7 were used as screening tools, and results regarding diagnostic accuracy indicate that these might be more suitable in an ED population [54]. Others have correspondingly reported good reliability and validity for the PHQ-9 in the ED [55]. Another conceivable explanation for the longitudinal improvement in mental health symptom burden in our cohort could be rooted in mental health conditions going along with and aggravating somatic symptoms like breathlessness [56]. Thus, acute episodes of depression or anxiety might have played a role in triggering respiratory ED consultations, and remission over time [57] could have caused part of the trends seen. It must be noted in this regard that PHQ-4 scores at follow-up in our population were higher than reported from other evaluations of longitudinal trends in unselected medical ED patients [58], which may also reflect the specific nature of respiratory symptoms [59].

Life satisfaction ratings seem to be more constant, being less health-related and thus presumedly entailing a lesser potential for situational distortion. In our analysis of the whole EMANet cohort, life satisfaction also appeared a rather stable construct less affected by acute circumstances [49]. Ratings also correspond in dimension to representative evaluations of the general population [19]. Furthermore, consistency of life satisfaction ratings over time does support the impression of ED visits not constituting a clear sentinel for deterioration of health for most of our population.

#### Determinants and implications of dropout

Survey non-response is frequently not at random but associated with patient characteristics and health-related outcomes [60, 61], and the demographic and medical diversity of ED consulters may promote disproportional susceptibility to attrition. Certain groups have been identified by others as tendentially more prone to drop out of longitudinal studies: patients with low socioeconomic and educational status, members of ethnic minorities, as well as patients with mental health problems [62–65]. This corresponds to lower education status and migration history identified as determinants of dropout in our study. Concerning education, this association did only show in the regression analysis, which underscores the importance of investigating dropout by multivariable methods. Reasons for selective non-response of migrants have been discussed, conceivable explanations encompassing language barriers and difficulties to be reached [66]. Loss of tourists to follow-up is also not surprising, as such tendentially younger and more mobile patients may have utilized the ED more “casually”, as the most obvious place to turn to when ill while on travel, for lack of knowledge about conceivable alternative options. Such consulters could potentially be less interested to take part in a study follow-up and might be more difficult to reach, with the additional issue of language barriers. On the other hand, presence of chronic conditions (as indicated by the multimorbidity variable) and attachment to a GP may characterize people for which the ED visit could have had larger impact on their health and health care situation. These may have been more motivated to take part in the follow-up survey. While the post-ED death rate in our study does not seem to be unusually high [67, 68], death has to be considered as a source of attrition bias as well, especially concerning data on HCU: very ill and/or old people who died prior to intended follow-up may also have been high-volume utilizers in the last period of their life [69]. This is inherently not represented in our data.

### Limitations

Firstly, we must stress that the results presented must be regarded with due interpretative caution considering the limited sample size of this follow-up study. While the dropout rate corresponds in dimension to other longitudinal survey studies following up ED visits [70, 71], potential attrition-related distortion of data is an issue of concern, as discussed above. We did investigate and present non-responder characteristics comprehensively and discussed potential distortions of results. Encouragingly, the influence of differential dropout on association estimates has been suggested as modest in several investigations [65, 72–74].

As to the retrospective nature of the follow-up survey, recall bias may also threaten validity. Such has been described as potentially considerable for HCU data [75]. While there is no consensus on optimal length of recall periods, better accuracy of self-reported HCU has been reported for shorter time frames, compared to e.g. yearly assessment [76]. Then again, tendential overreporting has been problematized for shorter periods [77].

Use of Likert scales for ED patient satisfaction is common [78], but they have been criticized as potentially skewed, low-dimensional and prone to ceiling effects [79]. By combining the satisfaction item with the question on benefit from the visit, our questionnaire intended to capture the whole spectrum of “good” or “bad” that subjectively comes out of a consultation, beyond process-oriented patient satisfaction. However, we must stress that this approach had an explorative character, as there currently is no established tool which integrates both satisfaction and outcome aspects. Existing validated instruments like the Consumer Emergency Care Satisfaction Scale (CECSS) [80] e.g. capture process aspects comprehensively, but do not include views on outcome. However, there are promising approaches: for patient-reported outcomes, Canadian researchers have recently published a validation study of a tool (PROM-ED) [81] aimed at capturing cardinal outcome domains identified from qualitative data [82].

Regarding the validity of patient-reported care satisfaction, the difference in ratings derived from telephone interviews vs. written questionnaires suggests that social desirability bias might be an important concern, dissatisfaction having been potentially more freely expressed in writing. Ratings of the visit as beneficial may be less affected by this bias source, as no differences could be noted. The discrepancy to satisfaction ratings underscores the notion that this survey item in fact captures a concept distinct from process-oriented satisfaction. As to validity, it must also be noted that this follow-up study had an explorative rather than confirmatory character. While validated scales and items were used if available (e.g. PHQ), many items were newly formulated on a theoretical basis, and we cannot be sure whether data sufficiently reflects the intended constructs. Additionally, some results could be spurious as to the problem of multiple univariable statistical testing. As to multivariable investigations, the models reported are also considerably more explorative and less supported by prior knowledge than e.g. clinical risk factor models. This calls for caution regarding any causal inference from these models. Interpretation of effect estimates of covariate factors is also problematic [83]. Potential bias resulting from missing data is a problem in many surveys as well as in secondary data analyses. Missingness in single variables was mostly well below 5%, but had cumulating effects in the multivariable models using listwise deletion which may have influenced results.

 Lastly, we must stress that our population consisted exclusively of patients with respiratory symptoms and results may reflect the specific nature of such (e.g., association of acute dyspnea with situational anxiety). The focus of recruitment on regular office hours also limits representativeness. Thus, inferences may not be readily generalizable to unselected ED patient populations.

## Conclusions

Our results indicate that most patients in this respiratory cohort are content with the ED visit and its outcome. Satisfaction with the care process could be improved by efforts to shorten waiting times and by working on seemingly trivial interpersonal aspects: friendly and respectful interaction, showing empathy with patients’ concerns, etc. However, patients are more than just customers [25], and thus subjective benefit from a consultation could be considered the more important outcome compared to a pleasant user experience.

In the debate concerning non-urgent ED visits, diversion to primary care is often proposed. Our data shows that self-assessment regarding the adequate care setting remains constant with distance of time to the ED visit. This suggests that patient judgments are rooted in more than situational anxiety. It would thus seem sensible for any efforts of redirecting utilizers to primary care to consider patients’ views and preferences adequately to assure acceptance. Patient-reported judgment however does not tell us whether primary care would have in fact been an appropriate alternative from a medical point of view.

ED consultations in our cohort do not indicate imperative deterioration of health in their aftermath, and life post-visit for many seems to go “back to normal”, especially if no underlying chronic illness is present. This may be symptom-specific however, as respiratory complaints do not always indicate serious disease.

Lastly, our study suggests that sex differences in perception of care and outcome may constitute a topic not yet studied sufficiently in health services research – in the ED and other settings. The trends suggested by our study should be further investigated in gender-focused ED research.

## Supplementary Information

**Additional file 1: Supplementary figure 1.** Forest plot of odds ratios from logistic regression model of rating ED visit as beneficial. **Supplementary table 1.** Self-reported general health, life satisfaction, and mental health measures, longitudinal comparison (baseline / follow-up).

## Data Availability

The datasets used and analyzed during this study are available from the corresponding author on reasonable request. Raw data is not deposited publicly, as this is not explicitly covered by participants’ informed consent.

## Abbreviations

ED: Emergency department
EMACROSS: Emergency and Acute Care for Respiratory Diseases beyond Sectoral Separation
MTS: Manchester Triage System
GP: General practitioner
EMS: Emergency medical services
EMANet: Emergency and Acute Medicine Network for Health Care Research Berlin
HCU: Health care utilization
PHQ: Patient Health Questionnaire
AUC: Area under the curve
ROC: Receiver operating characteristic
SD: Standard deviation
CASMIN: Comparative Analysis of Social Mobility in Industrial Nations
COPD: Chronic obstructive pulmonary disease
ICD: International Classification of Diseases
OR: Odds ratio
CI: Confidence interval
RTI: Respiratory tract infection
CT: Computed tomography
CECSS: Consumer Emergency Care Satisfaction Scale
PROM-ED: Patient-Reported Outcome Measure for Emergency Department Care
GAD: Generalized Anxiety Disorder Scale
EMASPOT: Systematic Screening for Comorbid Psychological Conditions in Cardiac ACSC Patients with Multimorbidity in the ED
