# Supplementary material for: Emergency department consultations for respiratory symptoms revisited: exploratory investigation of longitudinal trends in patients’ perspective on care, health care utilization, and general and mental health, from a multicenter study in Berlin, Germany
Source: BMC Health Serv Res. 2022 Feb 10;22:169. doi: 10.1186/s12913-022-07591-5 (PMC8830011; doi:10.1186/s12913-022-07591-5)
Supplement: Supplementary file 1 — Additional file 1: Supplementary figure 1. Forest plot of odds ratios from logistic regression model of rating ED visit as beneficial. Supplementary table 1. Self-reported general health, life satisfaction, and mental health measures, longitudinal comparison (baseline / follow-up). [file 12913_2022_7591_MOESM1_ESM.docx]

**Emergency department consultations for respiratory symptoms revisited: exploratory investigation of longitudinal trends in patients’ perspective on care, health care utilization, and general and mental health, from a multicenter study in Berlin, Germany**.

***Felix Holzinger, Sarah Oslislo, Lisa Kümpel, Rebecca Resendiz Cantu, Martin Möckel, Christoph Heintze***

**Additional file 1**

**Supplementary figure 1**

*Forest plot of odds ratios from logistic regression model of rating ED visit as beneficial*

**
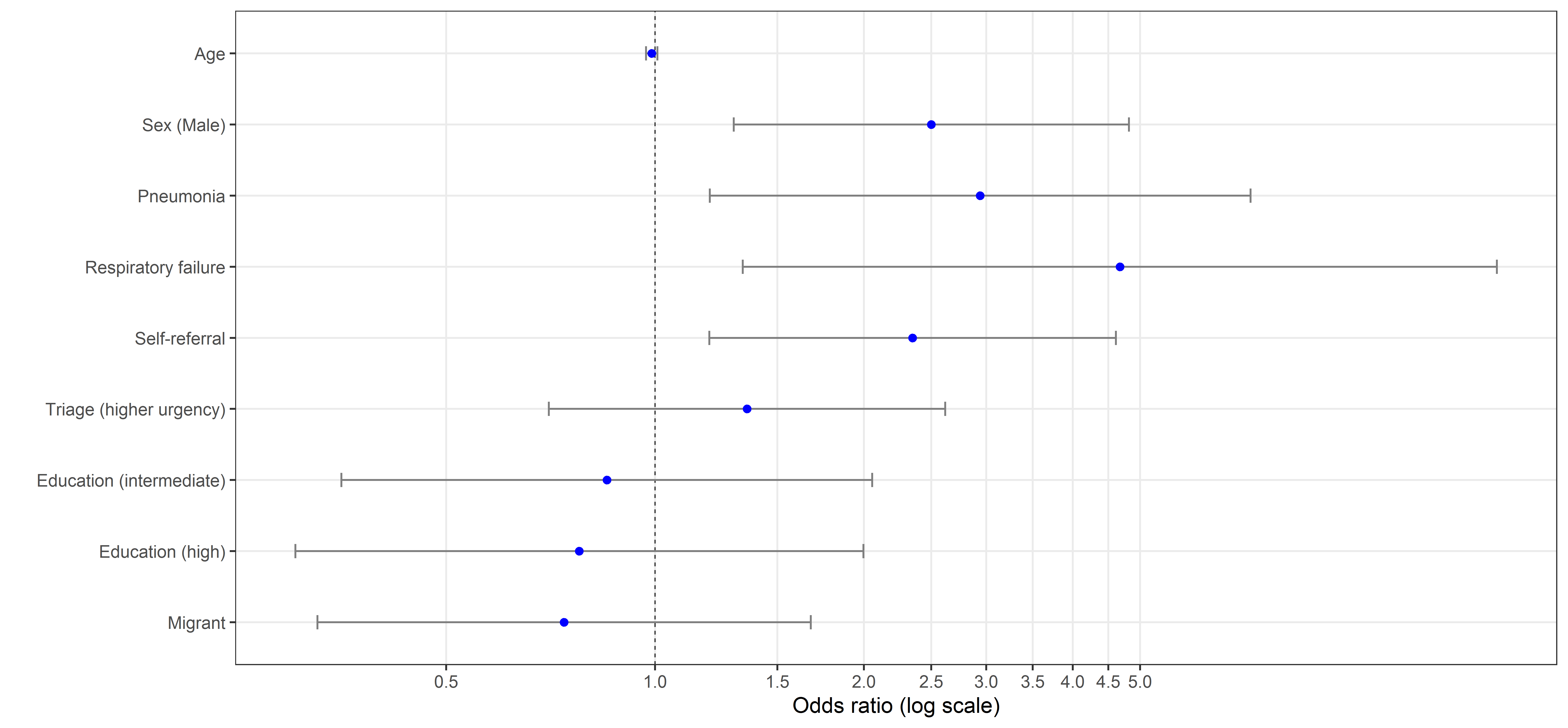
**

*Note*. Odds ratios represented on log scale for appropriate visualization of magnitudes of estimates and confidence intervals.

**Supplementary table 1**

*Self-reported general health, life satisfaction, and mental health measures, longitudinal comparison (baseline / follow-up)*

| Variable | n with data* | Mean baseline (SD) | Mean follow-up (SD) | p for pre-post comparison** |
| --- | --- | --- | --- | --- |
| **General health (0-100)** | 316 | 45.7 (24.9) | 61.5 (24.9) | <0.001 |
| **General life satisfaction (0-10)** | 308 | 7.1 (2.5) | 6.8 (2.5) | 0.092 |
| **PHQ-4 anxiety subscale** **(0-6)** | 309 | 1.6 (1.8) | 1.0 (1.4) | <0.001 |
| **PHQ-4 depression subscale (0-6)** | 313 | 2.1 (2.1) | 1.4 (1.8) | <0.001 |
| *Note.* *n = number of cases with baseline and follow-up data available; **Wilcoxon signed-rank test. | | | | |
